# Lipid metabolism in ferroptosis and ferroptosis-based cancer therapy

**DOI:** 10.3389/fonc.2022.941618

**Published:** 2022-08-01

**Authors:** Yonghao Sun, Zuoxing Xue, Tao Huang, Xiangyu Che, Guangzhen Wu

**Affiliations:** ^1^ Department of Urology, The First Affiliated Hospital of Dalian Medical University, Dalian, China; ^2^ Department of Urology, Dalian University Affiliated Xinhua Hospital, Dalian, China

**Keywords:** lipid metabolism, ferroptosis, tumor, tumor therapy, lipid peroxidation

## Abstract

Ferroptosis refers to iron-dependent, specialized, and regulated-necrosis mediated by lipid peroxidation, which is closely related to a variety of diseases, including cancer. Tumor cells undergo extensive changes in lipid metabolism, including lipid peroxidation and ferroptosis. Changes in lipid metabolism are critical for the regulation of ferroptosis and thus have important roles in cancer therapy. In this review, we introduce the characteristics of ferroptosis and briefly analyze the links between several metabolic mechanisms and ferroptosis. The effects of lipid peroxides, several signaling pathways, and the molecules and pathways involved in lipid metabolism on ferroptosis were extensively analyzed. Finally, our review highlights some ferroptosis-based treatments and presents some methods and examples of how these treatments can be combined with other treatments.

## 1 Feature of ferroptosis

The discovery of ferroptosis stemmed from the identification of systematic xCT in 1980 (1). It was not until 2012 that Dixon al. formally proposed the concept of ferroptosis (2). And ferroptosis is morphologically, biochemically and genetically different from other regulated cell death (RCD).

### 1.1 Morphological characteristics

The morphology of ferroptosis cells is significantly different from other RCD. Macroscopically, ferroptosis cells show swelling of the cytoplasm as well as organelles, deformation of the plasma membrane, and decreased chromatin condensation similar to necrosis (3, 4). Microscopically, ferroptosis cells show abnormalities in the mitochondrial structure, such as mitochondrial outer membrane rupture, an increase in mitochondrial membrane density, and reduced or even disappearance of the mitochondrial cristae.

### 1.2 Biochemical characteristics

**Figure 1:**
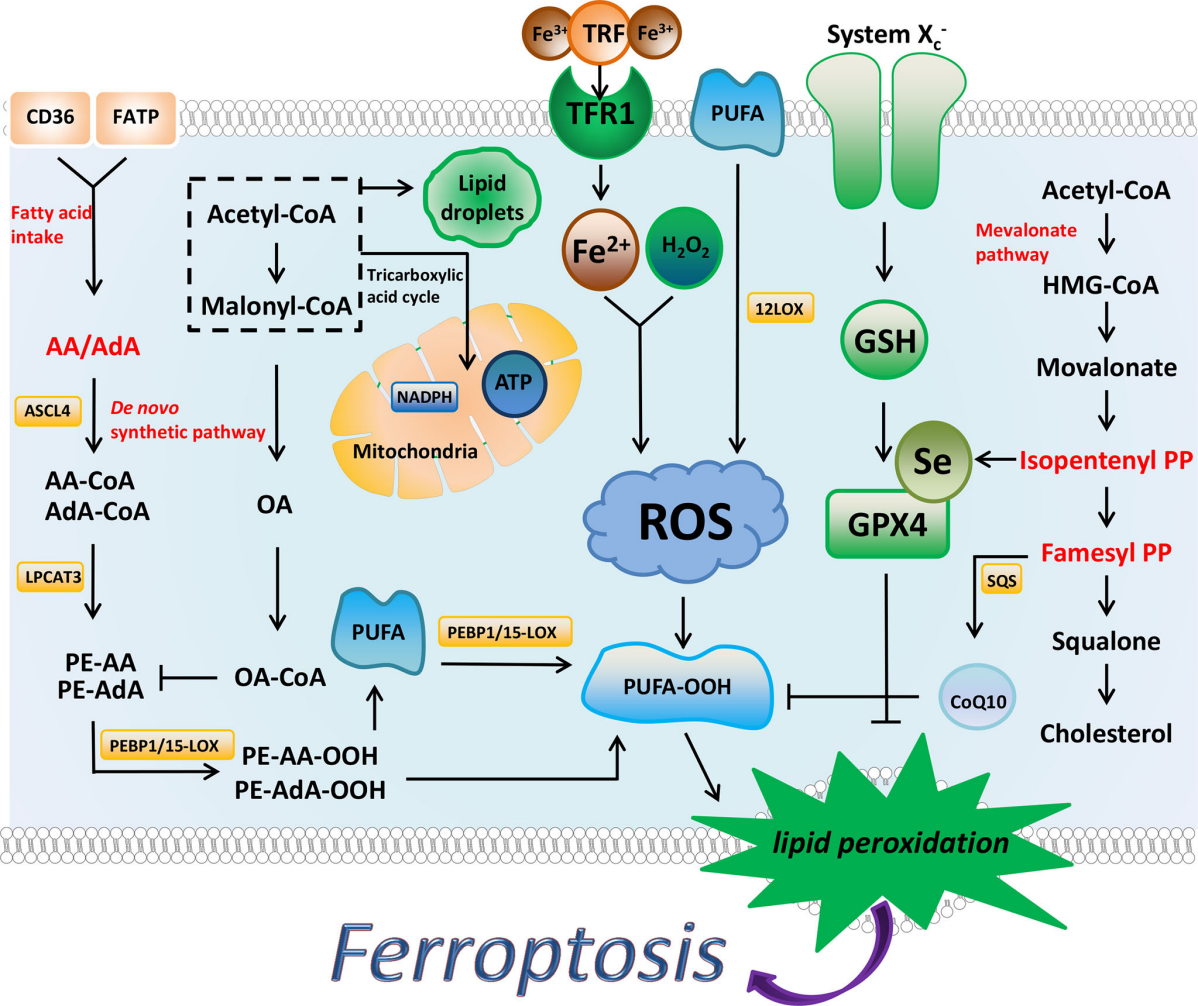
The regulatory substances and mechanisms of ferroptosis. Ferroptosis is driven by the accumulation of lipid peroxides. Iron metabolism, PUFA synthesis, and peroxidation promote the production of PUFA peroxides. AA and AdA are the key substances in the synthesis of PUFA. MUFA (such as OA) and Lipid droplets inhibit ferroptosis to a certain extent by affecting the synthesis of PUFA. NADPH has been shown to prevent lipid damage and counteract ferroptosis. System X_c_^-^/GSH/GPX4 axis and FSP1/DHODH/CoQ10 axis can inhibit ferroptosis through antioxidant effects. The synthesis process of cholesterol is closely related to these two antioxidant axes, and IPP and FPP are the key substances.

Abnormal iron and lipid metabolism in ferroptosis is obvious, iron is a key factor in lipid oxidation and ferroptosis, and abnormalities in its input, secretion, distribution, and storage can affect ferroptosis (5, 6). Mitochondria is an important organelle involved in the production of ROS (7), and lipid peroxides are type of ROS that directly induce ferroptosis (8). Normal cellular biological processes require the participation of ROS, but excessive ROS will destroy cellular components and lead to cell death (9). Two signaling axes, X_c_^-^/GSH/GPX4 and FSP1/DHODH/CoQ10 protect cells from ferroptosis. Specific regulatory factors and mechanisms are detailed below (**Figure 1**).

Current research shows that ferroptosis is inextricably linked to amino acid, glucose, iron, and lipid metabolism. Glutamate and glutamine involved in the amino acid pathway regulate ferroptosis (10). Cysteine input through system X_c_^-^ belongs to the amino acid pathway, and the transsulfurization pathway allows cells to convert methionine to cysteine. The mitochondrial TCA cycle and electron transport chain can promote cysteine deprivation-induced ferroptosis, the process involving amino acid metabolism, lipid metabolism, and glucose metabolism (11).

## 2 Lipid peroxidation and ferroptosis

Excessive accumulation of lipid peroxides is an important link in the process of ferroptosis, and substrates and enzymes involved in lipid peroxidation can regulate ferroptosis.

### 2.1 Key substrates and enzymes of lipid peroxidation

Ferroptosis is accompanied by the accumulation of lipid peroxides. Lipidomics analysis showed that PEs containing AA or epinephrine AdA in the membrane phospholipid family are susceptible to lipid peroxidation (12). Acyl-CoA synthetase long-chain family member 4 (ACSL4) and lysophosphatidylcholine acyltransferase 3 (LPCAT3) can acylate AA/AdA to membrane phospholipids, affect the properties of PUFA, and play important regulatory roles in ferroptosis (13, 14).

ACSL4 is an indispensable enzyme for lipid metabolism that catalyzes the binding of free long-chain fatty acids to CoA and preferentially recognizes arachidonic acid, and acylates it (15), after which LPCAT3 catalyzes the acylated AA into PL (14). The inhibition of these two enzymes can block the accumulation of lipid peroxides during ferroptosis, thus inhibiting ferroptosis to a certain extent. In addition, FSP1 catalyzes the conversion of ubiquinone to ubiquinol and prevents lipid peroxidation in the cell membranes (16).

### 2.2 Oxygenases in lipid peroxidation

Lipid peroxidation can be divided into enzymatic and non-enzymatic reactions (17), which the latter mainly refer to the entry of iron into cells through transferrin, followed by the Fenton reaction with hydrogen peroxide where excess oxygen free radicals are generated (18). Lipid oxidases involved in enzymatic reactions are divided into three types, namely lipoxygenase (LOX), cytochrome P450 oxidoreductase (POR), and cyclooxygenase (COX). The production of ROS requires the participation of these enzymes, furthermore, these enzymes are involved in ferroptosis (19). PUFA substrates are oxidized in the U-shaped fatty acid-binding channels of LOXs (20), the LOX gene family consists of six members: ALOX3, ALOX5, ALOX12, ALOX12B, ALOX15, and ALOX15B (21). TP53 is a tumor suppressor gene that suppresses the expression of SLC7A11 to deplete GSH, thus inducing ferroptosis (22). Different members of TP53 can induce ferroptosis *via* different ALOX (21, 23). Cytochrome P450 oxidoreductase (POR) can use electrons derived from the donor NADPH and provide them to downstream effectors (such as CYP and cytochrome p50) to reduce them. This causes the peroxidation of membrane polyunsaturated phospholipids which can lead to ferroptosis (24). COX-2 is upregulated during ferroptosis, however, inhibition of COX-2 does not modulate ferroptosis.

## 3 Lipid metabolism regulates ferroptosis

Compared with normal cells, the lipid metabolism of tumor cells is abnormal, and lipid metabolism is widespread in the process of ferroptosis. Fatty acid synthesis, fatty acid transport, the mevalonate pathway for cholesterol synthesis, and several major signaling pathways involved in lipid metabolism regulate ferroptosis.

### 3.1 PUFA and MUFA

PUFA play a unique and important role in regulating cell death, AA and AdA are the most critical substrate in ferroptosis. Hypermethylation of the promoter region results in low expression of ELOVL5 and FADS1 in intestinal-type gastric cancer (GC) cells, which are resistant to ferroptosis, and after supplementation with AA, the cells become ferroptosis-sensitive (25).

Treatment of cells with exogenous MUFA inhibits ferroptosis, and this protective effect is dependent on the involvement of ACSL3 (26). The mechanism is associated with the inhibition of ROS accumulation in the plasma membrane by MUFA, which competes with PUFA for binding to phospholipids. Increasing the ratio of saturated fatty acids (SFA) to monounsaturated fatty acids (MUFA) in cells, leads to lipotoxicity and apoptosis (27). The mechanism by which MUFA inhibits ferroptosis is distinct from its mechanism of inhibiting lipotoxicity by directing SFA into the triglyceride pool to form lipid droplets, which ultimately protects cells from lipotoxicity (28).

### 3.2 *De novo* synthetic pathway

Normal cells rely on glucose as their primary energy source, and excess glucose is used for *de novo* lipogenesis. SFA and MUFA can be produced through this pathway, while PUFA level is dependent on dietary intake (29), glucose in cancer cells is used for anabolism rather than for generating oxidative energy (30). Approximately 95% of the fatty acids in tumor cells are derived from endogenous synthesis (31, 32). *De novo* synthesized fatty acids are stored in neutral lipids (stored in the LD) and phospholipids (in the membrane), and lipid synthesis is exuberant in cancer cells. Lipid droplets act as potential scavengers of ROS and promote *de novo* lipid synthesis in cancer cells (33, 34), the presence of lipid droplets inhibits cell ferroptosis to a certain extent.

### 3.3 Fatty acid intake

In addition to *de novo* synthesis of fatty acids, fatty acid transporters such as fatty acid translocase (FAT/CD36), fatty acid transporter (FATP), and fatty acid-binding protein (FABP) can transport extracellular fatty acids into the cells (35). Similar to normal cells, cancer cells utilize exogenous lipids when *de novo* synthesis is inhibited (36).

The cluster of differentiation 36 (CD36) can be involved in fatty acid intake, and increased CD36 expression can lead to tumor metastasis. Blocking CD36 can inhibit tumor growth and metastasis in prostate cancer models (37). Fatty acid taken *via* CD36 is mostly stored rather than used for fatty acid oxidation, which may promote ferroptosis in CD36-overexpressing tumor cells (38). On the other hand, CD36 can suppress ferroptosis by exporting AA (39).

Besides CD36, FATP2 is also responsible for mediating fatty acid uptake. Selective inhibition of FATP2 can delay tumor progression, while loss of FATP2 leads to impaired uptake of AA, making cells resistant to ferroptosis (40). FATP2 expression was reduced in GC cells, but the cells enhanced their ability to synthesize fatty acids through *de novo* fatty acid synthesis so that AA deficiency had minimal effect, allowing cells to remain sensitive to ferroptosis (25).

### 3.4 Mevalonate pathway

Cholesterol can be oxidized and is an important component of the cell membrane structure and lipoprotein, studies have shown that cholesterol and ferroptosis have many links (41, 42). In addition to *de novo* synthesis of FAs and cholesterol by liver and adipocytes, cholesterol can also be synthesized *via* the mevalonate pathway, in which hydroxymethylglutaryl-CoA reductase (HMGCR) is the rate-limiting enzyme (43). Many enzymes associated with *de novo* synthesis and/or mevalonate pathway synthesis are thought to inhibit tumor growth (36, 44). The mevalonate pathway also affects selenoprotein biosynthesis (45), and GPX4 is a form of selenoprotein, thus, so the mevalonate pathway is related to ferroptosis. Statins have been used clinically for cholesterol-lowering therapy since their development (46), and they were later found that patients taking statins were resistant to some tumors, such as breast cancer (47). Statins can inhibit HMG-CoA reductase (HMGCR), resulting in the inactivation of GPX4 and induction of ferroptosis in mesenchymal cancer cells (48). FIN56 is a class III FIN that degrades GPX4 and activates squalene synthase (SQS), which can lead to the exhaustion of coenzyme Q10 (CoQ10), leading to ferroptosis (49), moreover, its mechanism of action involves the mevalonate pathway.

### 3.5 Signaling pathways regulate ferroptosis

In addition to GPX4 and FSP1, there are also several signaling pathways involved in lipid metabolism that can regulate ferroptosi**s**: the Hippo pathway involved in cell proliferation and regulation of cell size, the AMPK signaling involved in energy metabolism, and the HIF pathway involved in hypoxic conditions (50, 51). The regulatory mechanisms of these three pathways and their involvement in lipid metabolism are summarized in **Table 1**.

**Table 1:** This table introduces the three pathways that regulate ferroptosis, and some of their specific mechanisms, and states whether lipid metabolism processes are involved.

Signaling pathway	Mechanism	Involved in lipid metabolism	Refs
E-cadherin-NF2-Hippo-YAP	Downstream effector TAZ alters cell density	no	(52, 53)
	YAP affects ferroptosis by regulating target genes ACSL4 and TfR1	yes	(54)
	Cadherin affects cell-to-cell contact and EMT by regulating the Hippo pathway	no	(55)
AMPK	Activation of AMPK inhibits ferroptosis by inhibiting lipid peroxidation	yes	(56)
	AMPK promotes ferroptosis by directly blocking system X_c_^-^ activity	yes	(57)
HIF-2α-HILPDA	HIF-2α-HILPDA axis selectively enriches polyunsaturated lipids by activating the expression of hypoxia-inducible factors	yes	(58, 59)

## 4 Ferroptosis-related cancer treatment

**Figure 2:**
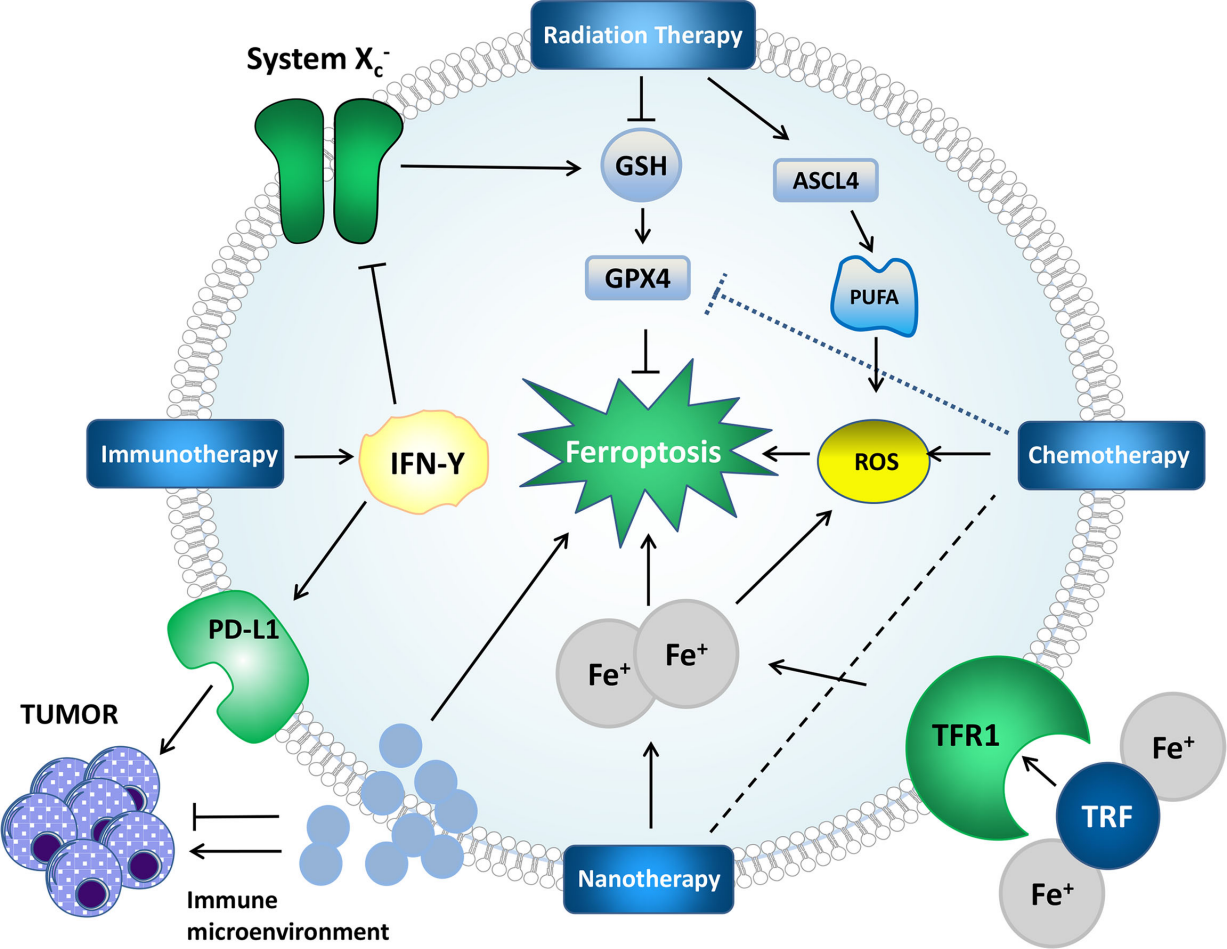
Mechanisms by which some treatments affect ferroptosis and tumors. Radiation therapy can generate ROS, induce lipid peroxidation, upregulate ASCL4, promote PUFA synthesis, provide a substrate for lipid peroxidation, deplete GSH and affect GPX4 to affect ferroptosis. IFN-γ in immunotherapy can affect system X_c_^-^, which further affects ferroptosis through the GPX4 pathway. PD-L1 can directly inhibit tumors. Nanotherapy can deliver iron and chemotherapeutic drugs to cells to achieve better therapeutic effects. The impact of the immune microenvironment on ferroptosis and tumors cannot be ignored.

RCD includes but is not limited to autophagy, pyroptosis, necroptosis, and ferroptosis. Signal transduction in RCDs can be artificially regulated and promote tumor cell death (60). The mechanisms of RCDs are different (61–63). Mechanistically, some drugs and compounds involved in ferroptosis function by regulating iron metabolism, lipid peroxidation, or both pathways. We summarize some ferroptosis-based treatments (**Table 2** and **Figure 2**).

**Table 2:** This table summarizes examples of radiation therapy, chemotherapy, immunotherapy, and other combination of treatments, and briefly describes the mechanism.

Therapy	Treatment	Combination drugs	Mechanism	Refs
Radiotherapy	RT	FINs	Up-regulates ACSL4, inhibits SLC7A11 or GPX4	(64)
Chemotherapy	Bortezomib	Iron	Increases intracellular iron content to induce ferroptosis	(65)
	doxorubicin and cisplatin	microRNA miR-133a	Targeted downregulation of ferritin light chain (FTL) protein	(66)
	Statins	–	Reduces selenoproteins (such as GPX4) and CoQ10 biosynthesis	(67)
	Cyst(e)inase	FINs	Depletes extracellular cystine	(16)
	Sorafenib	siRNA	Inhibits the system X_c_^-^	(68)
Immunotherapy	PD-L1 inhibitors	FINs	Releases IFN-γ to reduce the uptake of cystine	(69)
	photodynamic therapy	–	Increases the level of lymphocyte infiltration in tumors and recruits immune cells to secrete IFN-γ	(70)
Nanotherapy	Nanoparticle materials	Iron	Increases intracellular iron content and activates the Fenton response	(71)
		Sorafenib and Cisplatin	Increases the sensitivity of cancer cells to drugs	(72, 73)
		PUFA	Regulates lipid peroxidation	(74, 75)
	exosomes	erastin	Avoids adverse reactions	(76)

### 4.1 Ferroptosis-based drug therapy

#### 4.1.1 Iron metabolism-related drug therapy

There is widespread dysregulation of iron metabolism in well-known cancer types, such as breast cancer, pancreatic cancer, lymphoma, hepatocellular carcinoma, glioblastoma, prostate cancer, and colorectal cancer (77). Regulation of ferroptosis *via* iron metabolism is a reliable approach for the treatment of different cancer types. Artesunate enhances the lysosomal activity and increases the lysosomal iron concentration in cells (78). Furthermore, it regulates the mRNA expression levels of iron-related genes (79), the expression of NCOA4 to promote ferritin phagocytosis to increase the cellular iron concentration, and other processes which can induce ferroptosis (80). Artesunate regulates iron metabolism through the above-mentioned mechanisms and induces ferroptosis in pancreatic cancer cells (81). Artesunate also inhibits Burkitt lymphoma by inducing ferroptosis (82). Dihydroartemisinin increases the autophagic degradation of ferritin by accumulating in lysosomes, increasing the cellular iron content, and inducing ferroptosis (78, 83), thereby triggering ferroptosis in glioma cells (84). Cisplatin, which is involved in ferritin phagocytosis and leads to an increase in cellular free iron levels, also induces ferroptosis (85). In a cisplatin-resistant head and neck cancer cell model, the treatment effect of artesunate was poor, and the inhibition of the NRF2-ARE pathway increased the sensitivity of artesunate and reversed the ferroptosis resistance of the model (86). Changes in iron metabolism were also involved in this process.

#### 4.1.2 Lipid peroxidation-related drug therapy

The two signal axes mentioned earlier, system X_c_^-^/GSH/GPX4 axis and FSP1/DHODH/CoQ10 axis, as well as transcription factor NERF2, p53 gene, and three types of lipid oxidase: COX, cytochrome POR, and LOX, can serve as targets for regulating lipid peroxidation. In terms of regulating the lipid peroxidation pathway, gemcitabine is the main treatment drug for Pancreatic ductal adenocarcinoma (PDAC), which leads to the accumulation of ROS and induces ferroptosis when it exerts its therapeutic effect (87). Inhibition of the HSPA5-GPX4 pathway can induce and provoke ferroptosis in PDAC cells and enhance the anticancer effect of gemcitabine (88). Sorafenib can directly inhibit the function of system X_c_^-^ and activate the NRF2-SLC7A11 signaling pathway. The use of the NRF2 inhibitor trigonelline in a sorafenib-treated mouse model was found to promote ferroptosis and enhance the tumor suppressor effect of sorafenib (68). In a glioblastoma multiforme (GBM) model, temozolomide (TMZ) induces SLC7A11 expression by activating NRF2 and ATF4, which partially inhibits ferroptosis (89).

### 4.2 Combining ferroptosis with other treatments

#### 4.2.1 Radiation therapy

Radiation therapy can induce various types of DNA damage in cells (90) and produce highly reactive OH radicals and other ROS by stimulating oxidase activity (91, 92), making it capable of inducing ferroptosis. The ionizing radiation produced by radiotherapy induces ferroptosis in three ways (93): 1. Production of excess ROS to induce lipid peroxidation. 2. Up-regulation of ACSL4 expression which further promotes PUFA-PL biosynthesis. 3. GSH consumption inhibits the protective effects of GPX4 on ferroptosis. The combination of radiation therapy and ferroptosis inducers such as erastin and sulfasalazine (SAS) for class I FIN, RSL3 and ML162 for class II FIN, and FIN56 for class III FIN increases tumor sensitivity to radiation therapy. Studies have shown that these ferroptosis inducers combined with radiation therapy increase the sensitivity of non-small cell lung cancer cells to radiation therapy and achieve better therapeutic effects than conventional radiotherapy (64). Recent studies have shown that the combination of class I FIN and radiotherapy for SLC7A11 has a better effect, while class II and class III FIN have poor pharmacokinetic effects and are prone to cytotoxicity in actually combined therapy, and the overall therapeutic effect is not as good as that of class I FIN in combination with radiation therapy (92).

#### 4.2.2 Chemotherapy

At present, based on iron and lipid metabolism, two important factors related to ferroptosis, some effective drugs and methods for reversing tumor resistance have been discovered. Inhibition of ferroptosis-related genes and pathways in tumor cells is thought to be one of the reasons for drug resistance (94, 95).

Regarding iron metabolism, initial studies have shown that TFR is associated with tumor drug resistance, and down-regulation of TFR can reverse tumor drug resistance (96). Ferritin levels in multiple myeloma cells are thought to be directly related to bortezomib resistance, iron supplementation in multiple myeloma cell lines increases cell death, and ferritin reduction allows tumor cells to overcome bortezomib resistance (65). After targeted down-regulation of the ferritin L subunit by microRNA miR-133a in cisplatin and doxorubicin-resistant breast cancer cells, the cells showed increased sensitivity to these drugs (66).

Cells with high expression of GSH and the light chain subunit xCT in system X_c_^-^ exhibit resistance to drugs and radiation *via* lipid metabolism-related pathways (97, 98). The drug resistance of tumor cells is believed to originate from its dependence on GPX4, and inhibition of GPX4 can induce ferroptosis and attenuate the drug resistance of tumor cells (99). FSP1 acts as an oxidoreductase in the plasma membrane to reduce CoQ and inhibit lipid peroxidation and ferroptosis, a pathway thought to be parallel to the GSH/GPX4 axis (16), this pathway also shows the potential of anti-tumor drug resistance (100): in a mouse model established using ferroptosis-resistant H460 lung cancer cell xenografts, the inhibition of FSP1 resulted in significant tumor suppression in both GPX4 KO and GPX4 KO/FSP1 KO groups (16).

#### 4.2.3 Immunotherapy

The role of ferroptosis in regulating tumor immunity is bidirectional. It not only kills tumors, but also upregulates immune checkpoints and the production of immunosuppressive mediators can promote tumor growth. Interferon-gamma (IFN-γ) released from CD8^+^ T cells downregulates the expression of SLC3A2 and SLC7A11 in system X_c_^-^, leading to ferroptosis in mouse model tumor cells (69). Another study reported that IFN-γ secreted by CD8^+^ lymphocytes upregulates PD-L1 in ovarian cancer cells, promoting tumor growth (101). Therefore, it is speculated to imagine that the combination of immune checkpoint inhibitors and ferroptosis therapy may have a notable effect. TAMs are M2 polarized macrophages that secrete vascular endothelial growth factor (VEGF) to promote tumor growth (102). IFN-γ inhibits TAM differentiation in the tumor microenvironment and converts TAMs to M1 macrophages (103, 104), M1 macrophages are more resistant to ferroptosis than M2 macrophages, and studies have shown that nitric oxide synthase (iNOS)/NO regulates macrophage ferroptosis resistance, and decreased levels of iNOS result in decreased ferroptosis resistance in M1 cells, whereas NO donors increase ferroptosis resistance in M2 cells (105). Photodynamic therapy can increase tumor lymphocyte infiltration and recruit immune cells to secrete IFN-γ and enhance ferroptosis (70), which involves complex regulation of the immune microenvironment. The combination of ferroptosis and immunotherapy has broad prospects in the future, but the complexity of the immune microenvironment of tumor cells, the positive and negative effects of ferroptosis in tumor regulation, and the toxicity of ferroptosis-inducing agents on organisms require further in-depth experiments and inquiry.

#### 4.2.4 Nanotherapy

Ferroptosis-based nanotherapeutics are currently under development, nanomaterials are generally divided into iron-based and non-iron-based nanomaterials. Iron-containing nanoparticulate materials can release iron to the tumor site and trigger the Fenton reaction to generate ROS, which induces ferroptosis (71). Chemotherapeutic drugs or ferroptosis inducers can be loaded into iron-based nanoparticles, such as sorafenib and cisplatin, to work more effectively (72, 73). Non-iron-based nanomaterials are iron-free in the first place, and they can function by carrying ferroptosis-inducing agents, carrying lipids, carrying non-coding RNAs, etc (106). PUFA can be supplemented by nanoparticle drugs, which can modulate lipid peroxidation and induce ferroptosis in tumor cells (74, 75). Furthermore, erastin has poor water solubility and is nephrotoxic, folic acid-labeled erastin-loaded exosomes were used in triple-negative breast cancer cells to avoid nephrotoxicity, and the treatment produced a good therapeutic effect (76). Tumor cell secretion of exosomal PD-L1 suppresses T cell activity, blocks immune checkpoints, and may lead to resistance to therapy (107). Nanounits constructed from exosome inhibitors and ferroptosis inducers can link the immunogenic advantage of exosome inhibition with ferroptosis, this is a new and effective immunotherapy strategy (106).

## Conclusion and opinion

There is a wide range of metabolic abnormalities associated with ferroptosis. This abnormal regulation of metabolic pathways occurs in many diseases, especially cancer. Researchers have discovered some regulatory substances, pathways, genes, enzymes, and other components involved in ferroptosis. The presence of these target sites makes it possible to achieve disease treatment by regulating ferroptosis. In this review, we summarize and present the link between lipid metabolism and ferroptosis, with a focus on treatments that modulate ferroptosis by regulating lipid metabolism, and in combination with other treatments to achieve tumor therapy. Although the roles of many related pathways and factors have been demonstrated and validated in different cancer models, there are still some issues that have not yet been resolved.

Cancer treatment by regulating ferroptosis has been confirmed and applied in experimental processes and clinical environments. However, there are indeed differences in the effects of *in vitro* experiments and *in vivo* treatment. The side effects of some treatment methods also limit their practical application. For different types of cancer, targeted therapy should be selected according to their characteristics. Combining ferroptosis-based treatment with other treatment methods to promote strengths and avoid weaknesses appears to be a better treatment strategy in the future.

## Author contributions

XC and GW contributed to conception and design of the manuscript. YS wrote the first draft of the manuscript. YS, ZX and TH modified the manuscript. All authors contributed to the manuscript and approved the submitted version.

## Funding

The PhD Start-up Fund of Liaoning Province from GW (2021-BS-209, Liaoning Province, 30000 CNY). Dalian Youth Science and Technology Star from GW (2021RQ010, Dalian, 100000CNY); Liaoning Provincial Department of Education(LJKZ0860).

## Conflict of interest

The authors declare that the research was conducted in the absence of any commercial or financial relationships that could be construed as a potential conflict of interest.

## Publisher’s note

All claims expressed in this article are solely those of the authors and do not necessarily represent those of their affiliated organizations, or those of the publisher, the editors and the reviewers. Any product that may be evaluated in this article, or claim that may be made by its manufacturer, is not guaranteed or endorsed by the publisher.

## Abbreviations

AA, trioxyarachidonic acid; ACC, acetyl-CoA carboxylase; ACSL4, long-chain family member 4; ACSL3, acyl-CoA synthetase long-chain family member 3; AdA, adrenal acid; ALOX, Arachidonate lipoxygenase; AMPK, 5’adenosine monophosphate-activated protein kinase; ATP, adenosine triphosphate; BECN1, beclin 1; CD36, cluster of differentiation 36; CN-A, Cotylenin A; CoQ10, Coenzyme Q10; COX, cyclooxygenase; cPLA_2_, cytoplasmic phospholipase A2; CYP, Cytochrome P450; DHODH, dihydroorotate dehydrogenase; DPP4, dipeptidyl peptidase 4; ECM, extracellular matrix; ELOVL5, elongase of very-long fatty acid 5; EMP1, epithelial membrane protein 1; EMT, epithelial-mesenchymal transition; FABP: fatty acid binding protein; FADS1: fatty acid desaturase 1; FAT: fatty acid translocase; FATP2: fatty acid transporter 2; Fer-1, ferrostatin-1; FIN, ferroptosis inducers; FPP, farnesyl pyrophosphate FSP1; ferroptosis suppressor protein1; GBM, glioblastoma multiforme; GC, gastric cancer; GPX4, glutathione peroxidase 4; GSH, γ-L-glutamyl-L-cysteinylglycine; GSSG, glutathione disulfide; HIF, hypoxia-inducible factor; HILPDA, hypoxia-inducible, lipid droplet-associated protein; HSPA5, heat shock protein family A member 5 IFN-γ, Interferon gamma; IPP, isopentenyl pyrophosphate; KO, knock out; LD, lipid droplets; LOX, lipoxygenases; LPA, Lysophosphatidic Acid; LPCAT3, lysophosphatidylcholine acyltransferase 3; ML162, molecular libraries 162; MUFA, monounsaturated fatty acids; NADPH, nicotinamide adenine dinucleotide phosphate; NCOA4, nuclear receptor coactivator 4; NOS, nitric oxide synthase NADPH oxidase 4; NRF2, nuclear factor erythroid 2-related factor 2; PE, phosphatidylethanolamine; PDAC, pancreatic ductal adenocarcinoma; PD-L1, Programmed cell death ligand 1; PEITC, phenethyl isothiocyanate; PLINs, perilipins; PMN-MDSC, polymorphonuclear myeloid-derived suppressor cell; POR, cytochrome; PRRs, pattern recognition receptors P450 oxidoreductase; RCD, regulated cell death; ROS: reactive oxygen species; PUFA, polyunsaturated fatty acid; RSL3, Ras selective lethal 3; RSL5, Ras selective lethal 5; SAS, sulfasalazine; SFA, saturated fatty acids; SLC3A2, Solute carrier family 3 member 2; SLC7A11, Solute carrier family 7 member 11; SQS, squalene synthase; STAT3, signal transducer and activator of transcription 3; TAM, Tyro3, Axl, and Mer; TAZ, transcriptional co-activator with PDZ-binding motif; TCA, tricarboxylic acid cycle; TCRs, T cell receptors; TFR1, ferrotransferrin receptor 1; TMZ, temozolomide; TNFR, tumor necrosis factor receptor; TP53, tumor protein 53; TRF, transferrin; YAP, yes-associated protein 1; VEGF, vascular endothelial growth factor.

## Glossary

**Table:**

AA	trioxyarachidonic acid
ACC	acetyl-CoA carboxylase
ACSL4	long-chain family member 4
ACSL3	acyl-CoA synthetase longchain family member 3
AdA	adrenal acid
ALOX	Arachidonate lipoxygenase
AMPK	5’adenosine monophosphate-activated protein kinase;
ATP	adenosine triphosphate
BECN1	beclin 1
CD36	cluster of differentiation 36
CN-A	Cotylenin A
CoQ10	Coenzyme Q10
COX	cyclooxygenase
cPLA2	cytoplasmic phospholipase A2
CYP	Cytochrome P450
DHODH	dihydroorotate dehydrogenase
DPP4	dipeptidyl peptidase 4
ECM	extracellular matrix
ELOVL5	elongase of very-long fatty acid 5;
EMP1	epithelial membrane protein 1
EMT	epithelial-mesenchymal transition
FABP	fatty acid binding protein
FADS1	fatty acid desaturase 1
FAT	fatty acid translocase
FATP2	fatty acid transporter 2
Fer-1	ferrostatin-1
FIN	ferroptosis inducers
FPP	farnesyl pyrophosphate
FSP1	ferroptosis suppressor protein1
GBM	glioblastoma multiforme
GC	gastric cancer
GPX4	glutathione peroxidase 4
GSH	g-L-glutamyl-Lcysteinylglycine;
GSSG	glutathione disulfide
HIF	hypoxia-inducible factor;
HILPDA	hypoxia-inducible lipid droplet-associated protein
HSPA5	heat shock protein family A member 5 IFN-γ Interferon γ
IPP	isopentenyl pyrophosphate
KO	knock out
LD	lipid droplets
LOX	lipoxygenases
LPA	Lysophosphatidic Acid
LPCAT3	lysophosphatidylcholine acyltransferase 3;
ML162	molecular libraries 162
MUFA	monounsaturated fatty acids;
NADPH	nicotinamide adenine dinucleotide phosphate
NCOA4	nuclear receptor coactivator 4
NOS	nitric oxide synthase NADPH oxidase 4
NRF2	nuclear factor erythroid 2-related factor 2
PE	phosphatidylethanolamine;
PDAC	pancreatic ductal adenocarcinoma
PD-L1	Programmed cell death ligand 1
PEITC	phenethyl isothiocyanate
PLINs	perilipins
PMN-MDSC	polymorphonuclear myeloid-derived suppressor cell
POR	cytochrome;
PRRs	pattern recognition receptors P450 oxidoreductase
RCD	regulated cell death
ROS	reactive oxygen species
PUFA	polyunsaturated fatty acid;
RSL3	Ras selective lethal 3
RSL5	Ras selective lethal 5
SAS	sulfasalazine
SFA	saturated fatty acids
SLC3A2	Solute carrier family 3 member 2;
SLC7A11	Solute carrier family 7 member 11
SQS	squalene synthase;
STAT3	signal transducer and activator of transcription 3
TAM	Tyro3
Axl	and Mer
TAZ	transcriptional co-activator with PDZ-binding motif;
TCA	tricarboxylic acid cycle
TCRs	T cell receptors
TFR1	ferrotransferrin receptor 1
TMZ	temozolomide
TNFR	tumor necrosis factor receptor;
TP53	tumor protein 53
TRF	transferrin
YAP	yes-associated protein 1;
VEGF	vascular endothelial growth factor.
